# Structural and Pharmacological Effects of Ring-Closing Metathesis in Peptides

**DOI:** 10.3390/molecules15096638

**Published:** 2010-09-21

**Authors:** Øyvind Jacobsen, Jo Klaveness, Pål Rongved

**Affiliations:** School of Pharmacy, University of Oslo, Sem Sælands vei 3, P. O. Box 1068 Blindern, 0316 Oslo, Norway; E-Mails: jo.klaveness@farmasi.uio.no (J.K.); pal.rongved@farmasi.uio.no (P.R.)

**Keywords:** ring-closing metathesis, peptides, structure, pharmacological properties

## Abstract

Applications of ring-closing alkene metathesis (RCM) in acyclic α- and β-peptides and closely related systems are reviewed, with a special emphasis on the structural and pharmacological effects of cyclization by RCM.

## Table of Contents

1.   Introduction                                                2

2.   Synthesis of Peptidic RCM Precursors         4

3.   Catalysts, Solvents, Reaction Conditions         5

4.   C^α^(*i*)→C^α^(*i*), m = 4, (Z), n = 5 cyclizations         6

5.   C^α^(*i*)→C^α^(*i+1*), m = 4, (Z)/H_2_, n = 8 cyclizations         6

6.   C^α^(*i*)→N(*i+1*), m = 4-6, (Z), n = 7-9 cyclizations         7

7.   C^α^(*i*)→CO(*i+1*), m = 10, H_2_, n = 15 cyclizations         8

8.   C^α^(*i*)→C^α^(*i+2*), m = 4, (E)/(Z), n = 11 cyclizations         8

9.   C^α^(*i*)→C^α^(*i+2*), m = 6, H_2_, n = 13 cyclizations         9

10. C^α^(*i*)→C^α^(*i+2*), m = 8, (E)/(Z), n = 15 cyclizations         9

11. C^α^(*i*)→C^α^(*i+2*), m = 10, (E)/(Z)/H_2_, n = 17 cyclizations         11

12. C^α^(*i*)→C^α^(*i+2*), m = 16, (E)/(Z), n = 23 cyclizations         13

13. N(*i*)→CO(*i+2*), m = 7, (E)/H_2_, n = 16 cyclizations         13

14. C^α^(*i*)→C^α^(*i+3*), m = 4, (E)/(Z)/H_2_, n = 14 cyclizations         14

15. C^α^(*i*)→C^α^(*i+3*), m = 5, (E)/(Z)/H_2_, n = 15 cyclizations         17

16. C^α^(*i*)→C^α^(*i+3*), m = 8, (E)/H_2_, n = 18 cyclizations         18

17. “N”(*i*)→C^α^(*i+3*), m = 3 and 4, (E)/(Z), n = 13 and 14 cyclizations         19

18. N(*i*)→C^α^(*i+3*), m = 13, (E), n = 24 cyclizations         20

19. N(*i*)→CO(*i+3*), m = 7, (E)/(Z)/H_2_, n = 19 cyclizations         20

20. N(*i*)→N(*i+3*), m = 7 and 8, (E)/(Z), n = 17 and 18 cyclizations         21

21. C^β^(*i*)→C^β^(*i+3*), m = 8, (E)/H_2_, n = 21 cyclizations         22

22. C^α^(*i*)→C^α^(*i+4*), m = 6, (E)/(Z)/H_2_, n = 19 cyclizations         22

23. C^α^(*i*)→C^α^(*i+4*), m = 8, (Z), n = 21 cyclizations         23

24. C^α^(*i*)→C^α^(*i+4*), m = 8 and 10, (E)/(Z)/H_2_, n = 21 and 23 cyclizations         24

25. N(*i*)→C^α^(*i+4*), m = 6, (E)/H_2_, n = 20 cyclizations         26

26. CO(*i*)→N(*i+4*), m = 2, (E)/(Z)/H_2_, n = 13 cyclizations         27

27. C^α^(*i*)→C^α^(*i+5*), m = 4, (E)/(Z)/H_2_, n = 20 cyclizations         28

28. C^α^(*i*)→C^α^(*i+7*), m = 8-12, (E)/(Z)/H_2_, n = 30-34 cyclizations         31

29. Conclusions                                                32

References and Notes                                                33

## 1. Introduction 

Olefin metathesis occupies a prominent place in contemporary synthetic organic chemistry and has been extensively reviewed by Grubbs [1], Nicolaou [2] and Perez-Castells [3], among others. Since the pioneering work in 1995 by Miller and Grubbs [4] ring-closing metathesis (RCM) has seen widespread application in the fields of peptides and peptidomimetics. The first review by Gracias and Aube was published even the same year [5]. In 2008 Brik reviewed metathesis in general in peptides and peptidomimetics [6], but since then many new examples of the application of metathesis in general, and RCM in particular, to peptides and peptidomimetics have appeared. Importantly, RCM has been applied to a wide range of biologically active peptides. The focus of this review is on structural and pharmacological aspects of RCM in peptidic systems and, where such information is available, on the correlation of the two. In particular, we review what is known about the structural and pharmacological effects of installing an intramolecular RCM-derived bridge in peptidic compounds by comparing the structural and pharmacological properties of the cyclic compounds to those of their acyclic analogues. 

The term “structure” could mean the instantaneous structure, the thermally accessible conformations and their occupancies, an average structure, or a minimum energy, solid state or “static” structure. Different experimental techniques are applicable under different conditions and in different situations and probe different time domains and thus different aspects of structure. Whereas X-ray diffraction is very useful to determine static structures in the crystal state, NMR and CD spectroscopy provide information on the thermal ensemble of conformations and their (relative) occupancies in solution phase. In this review the different meanings of the term “structure” are used depending on what experimental technique has been applied in each case. 

By “pharmacological properties” we mean all relevant pharmacokinetic and pharmacodynamic properties of a potential drug candidate. However, the emphasis is on the effects of macrocyclization by RCM on affinity or potency (e.g., as measured by dissociation constants, IC_50_ values *etc*.), receptor selectivity, protease lability and metabolic stability *in vivo.*

**Figure 1:**
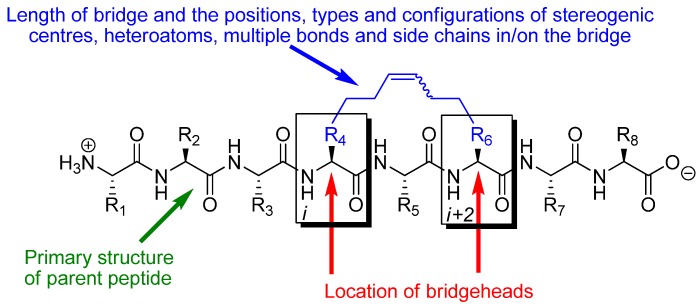
The structural and pharmacological effect of installing an RCM-derived bridge in a peptidic system is dependent on the primary structure of the parent peptide, the length of the bridge, the positions, types and configurations of stereogenic centres, heteroatoms, multiple bonds and side chains in/on the bridge, and the relative and absolute positioning of the bridgeheads along the backbone. If the bridgehead atoms are α-carbons the stereochemistry at the bridgehead atoms is also of significance.

The effect of RCM in a peptidic system depends on the conformational and pharmacological properties of the particular substituted macrocycle that results from RCM and how these compare with the conformational and pharmacological properties of the parent peptide. The parameters determining the effect of RCM in a peptidic system are thus: 1) the number (***m***) of atoms in the RCM-derived bridge, counted along the shortest path from bridgehead to bridgehead, 2) the positions and types of heteroatoms, multiple bonds, stereogenic centres and side chains in and on the bridge, 3) the configurations of stereogenic centres and multiple bonds, in particular the RCM-derived double bond in the bridge (note, however, that the double bond often is reduced by catalytic hydrogenation), 4) what peptide the bridge is installed in, *i.e.* what the primary structure of the parent peptide is, 5) which atoms (C^α^, C^β^, N or the carbonyl carbon) in the backbone of the parent peptide that serve as bridgeheads, and 6) their relative and absolute positions in the peptide, *i.e.* on which residues *i* and *i+k* ($i𝜖\mathbb{N},k𝜖{\mathbb{N}}_{0}$) they are located (Figure 1). In addition, when one or both of the bridgeheads are α-carbons the stereochemistry at the bridgeheads is also a factor.

The great number and diversity of peptidic systems in which RCM has been applied makes it difficult to classify the reported examples according to primary structure characteristics or biological effect in a unique way. In this review the material is therefore organized according to which atom types that serve as bridgeheads, the relative positioning of the bridgeheads in the peptide backbone and the length (***m***) of the RCM-derived bridge. In addition, the chapter headings indicate the stereochemistry of the RCM-derived double bond in the bridge and/or whether the corresponding hydrogenated compound has been studied. All descriptors refer to the compounds that are discussed in the text. For a given peptide, when the positions, types and configurations of stereogenic centres, heteroatoms, additional multiple bonds and side chains in/on the bridge, and the absolute positioning of the bridge along the backbone, are also taken into account, these parameters determine which substituted macrocycle that results from installing an RCM-derived bridge, and vastly simplify the task of classifying all examples of RCM in peptidic systems. The total number of atoms in the resulting macrocycle (***n***), which is uniquely determined by the types of bridgeheads, their relative positioning and the length of the bridge (provided that it is specified whether the bridge is installed in an α- or β-peptide), is also given in each chapter heading. 

This review concentrates on acyclic α- and β-peptides and closely related systems and *monocyclic* analogues thereof synthesized by RCM. RCM in other peptide-like systems, e.g., in aza-peptides, where the backbone is fundamentally different from naturally occurring peptides, is not reviewed in this article. Because the focus of this review is on the structural and pharmacological *differences* between acyclic peptides and analogous peptides with an RCM-derived conformational constraint installed, less attention is given to papers describing the properties of only the cyclic peptides. There are a few examples of ring-closing alkyne metathesis in peptides in the literature. However, this review concentrates on ring-closing *alkene* metathesis, as this is the totally dominating metathesis modality in peptidic systems. Although the patent literature in this field has become very extensive, with 129 patent applications having been filed so far (31 May 2010), we have chosen to not refer to any of these, interesting as they may be, as they have not been subjected to peer review. Finally, total syntheses of natural products containing peptidic fragments employing RCM have also been left out. 

First, we briefly review some of the different methods by which an alkene moiety can be introduced into a peptide in order to set it up for RCM, and some of the most commonly used (pre)catalysts, solvents and reaction conditions for RCM in peptidic systems. 

## 2. Synthesis of Peptidic RCM Precursors

The two olefinic groups required for RCM can be introduced in a variety of ways. However, some methods stand out in the sense that they have been employed especially often. Most methods involve introducing the olefin as part of a protected amino acid building block, with subsequent assembly of an olefinic peptidic RCM precursor by standard peptide coupling techniques. An allyl group attached to an α-carbon atom can be introduced via commercially available (*R*)- or (*S*)-allylglycine. One-step *O*-allylation of *N*-protected serine [7,8] or tyrosine [9,10] and incorporation of the resulting building blocks into peptides by standard peptide coupling protocols have also been widely used. Additionally, asymmetric synthesis of olefinic *N*-protected monosubstituted and α,α-disubstituted α-amino acid building blocks with all-carbon side chains may be accomplished via diastereoselective alkylation of a glycine enolate equivalent derived from a chiral 5,6-diphenyloxazinone with alkyl halides [11]. 

In the field of β-peptides only *O*-allylation of β^3^-serine has seen application so far [12,13]. A further alternative is offered by Seebach’s direct allylation of amides by treatment with allyl bromide in the presence of the P4-phosphazene base [14]. 

Introduction of olefinic groups onto the N- or C-terminus of a peptide may be accomplished by acylation [15] or esterification/amidation with a suitable olefinic acid or alcohol/amine respectively. A C-terminal allylglycine can in principle also be installed by a peptide Claisen rearrangement of a C-terminal *O*-allylglycine residue [16]. The *O*-allylglycine residue can be incorporated by standard peptide coupling chemistry, or, as illustrated by Hebach and Kazmeier, via a 4-component Ugi reaction employing allyl isocyanoacetate as the isocyanide component [17].

## 3. Catalysts, Solvents, Reaction Conditions

**Figure 2:**
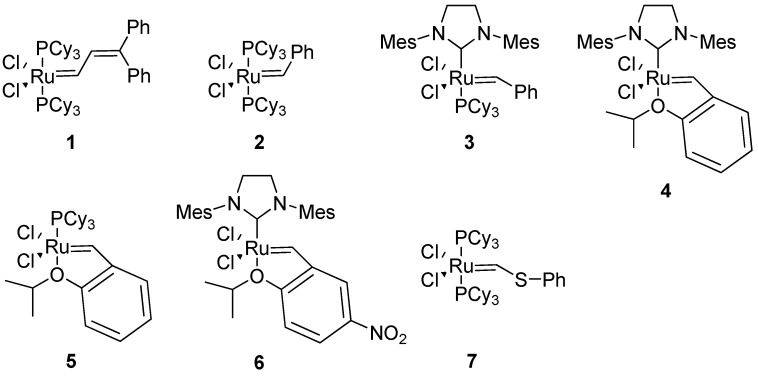
The most commonly used metathesis precatalysts for RCM in peptidic systems.

The exceptional functional group tolerance of the ruthenium (pre)catalysts **1-6** (Figure 2) appears to make these especially useful for RCM in a peptidic molecule, where heteroatoms and acidic protons are abundant. The catalyst **1** was used in the very first study of RCM in a peptidic system [4]. However, in later studies Grubbs’ 1st generation catalyst **2** and 2nd generation catalyst **3** have almost completely dominated the field. Typical precatalyst loadings have varied between 5-40 mol%, with 20 mol% having been relatively common [4,18].

For the cyclization of 3_14_-helical β^3^-peptides the 2nd generation Hoveyda-Grubbs precatalyst **4** gave very good results with respect to yields and almost complete (*E*)-selectivity [12]. Grubbs’ 2nd generation catalyst **3** in CH_2_Cl_2_ gave 4:1 (*E*)/(*Z*)-selectivity in a similar system [13].

From the very beginning of the field it was clear that RCM has to be done at low substrate concentration to minimize intermolecular oligomerization. In the first study by Miller and Grubbs a concentration of 0.002 M was employed [4]. This has remained a rather typical concentration. 

The by far most frequently used solvent has been CH_2_Cl_2_ [4]. A mixture of CH_2_Cl_2_ and DMF [4:1 (v/v)] was used in [19]. Addition of DMF allows a higher reaction temperature and is advantageous for peptides containing hydrophilic groups. In addition, it is compatible with on-resin cyclization. For the synthesis of hydrogen bond surrogate (HBS) helices, where high temperature is required, dichlorobenzene appears to be the preferred solvent [20,21]. In large-scale production of active pharmaceutical ingredients more environmentally benign solvents like toluene have been preferred [22].

The first example of on-resin RCM of a peptidic substrate was reported in 1996 by Grubbs and co-workers [9]. The beads were swollen in CH_2_Cl_2_, which is the solvent of choice for maximum activity of the metathesis (pre)catalysts. In some cases the resin loading has been quite high (0.34 mmol/g in reference [18]). For β^3^-peptides on-resin cyclization was facile using the 2nd generation Hoveyda-Grubbs catalyst **4** in a mixture [4:1 (v/v)] of TFE and CH_2_Cl_2_ [12], but not using Grubbs’ 2nd generation catalyst **3** in neat CH_2_Cl_2_ [13]. 

Microwave conditions have been shown to be beneficial in some cases. Notably, Marchán and co-workers observed much improved yields in on-resin cyclizations at 100 °C [18]. Microwave irradiation is thought to prevent aggregation and thus oligomerization. 

Arora and co-workers reported that only the 2nd generation Hoveyda-Grubbs catalyst **4** gives good yields with conventional heating in the synthesis of HBS helices from sterically hindered diolefins [20]. However, with microwave heating Grubbs’ 2nd generation catalyst **3** became much more active and the HBS helices can be isolated in good yields [21]. However, Grubbs’ 1st generation catalyst **2** and Ciba-ruthenium (CR) **7** do not afford the desired products even under microwave conditions.

In one case addition of chlorodicyclohexylborane was found to be very beneficial, increasing the yield in macrocyclizations from 20-40% to 80-90% [23]. The additive is thought to disrupt the nonproductive ruthenium chelate which may be formed when the olefin moiety is positioned close to a polar, coordinating group, e.g., a C-terminal methyl ester. 

## 4. C^α^(*i*)→C^α^(*i*), m = 4, (Z), n = 5 cyclizations

The *N*-formylated peptide *N*-For-Met-Leu-Phe-OH (fMLF) and its methyl ester (fMLF-OMe) are chemotactic agents that are highly specific for human neutrophils. Upon binding to neutrophil receptors like FPR and FPRL1 these peptides induce chemotaxis (directed migration of neutrophils along an increasing chemical gradient), phagocytosis, superoxide anion production and release of proteolytic enzymes, e.g., lysozyme. Lucente and co-workers synthesized analogues of fMLF-OMe by replacing Leu2 with α,α-diallylglycine and subjecting the resulting diolefinic peptide to RCM [24]. Both compounds were assayed for their ability to stimulate neutrophil chemotaxis. The cyclized compound was found to be more active than the diolefin precursor with respect to chemotactic index and superoxide anion production, but less active with respect to lysozyme release. Both compounds are, however, less active than the parent peptide fMLF-OMe. 

## 5. C^α^(*i*)→C^α^(*i+1*), m = 4, (Z)/H_2_, n = 8 Cyclizations

**Figure 3:**
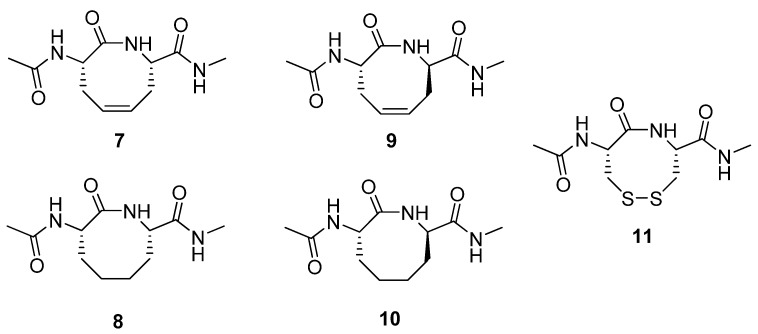
The (2*S*,7*R*)-alkene **9** adopts a type VIa β-turn conformation both in the crystal state and in aqueous solution.

β-turns are ubiquitous secondary structure elements in peptides and proteins. A complete β-turn is defined by four residues folding in such a way that the direction of the backbone changes by approximately 180° from the first residue to the last. There are eight subtypes of β-turns, each defined by a unique combination of dihedral angles for residues 2 and 3 in the turn [25,26]. A type VIa β-turn, defined by (φ,ψ)_i+1_ = (-60°, 120°) and (φ,ψ)_i+2_ = (-90°, 0°), and featuring a *cis* amide bond, is the β-turn with lowest abundance in proteins (< 1%) [25,26], but is nevertheless thought to be involved in several important biological processes, e.g., the thrombin-catalyzed cleavage of the V_3_ loop of HIV gp120 and substrate recognition of peptidyl prolyl isomerases [27,28]. The Reitz group prepared the constrained dipeptides **7-10** (Figure 3) by RCM and subjected several of them to structural scrutiny by NMR spectroscopy and X-ray diffraction [29]. The (2*S*,7*S*)-alkene **7** adopts a twist-boat-boat conformation in the crystal state, reminiscent of one of the low energy conformations of cyclooctadiene. The structure of its hydrogenated analogue **8** was found to be very similar to a previously studied disulfide analogue **11 [30]**. Interestingly, in contrast to **7** and **8**, the (2*S*,7*R*)-alkene **9** adopts a type VIa β-turn fold both in the solid state and in aqueous solution. This facilitates the formation of an intramolecular H-bond between the exocyclic acetamide groups. This type of *i*→*i+3* H-bond is characteristic of β-turns. 

## 6. C^α^(*i*)→N(*i+1*), m = 4-6, (Z), n = 7-9 Cyclizations

**Figure 4:**
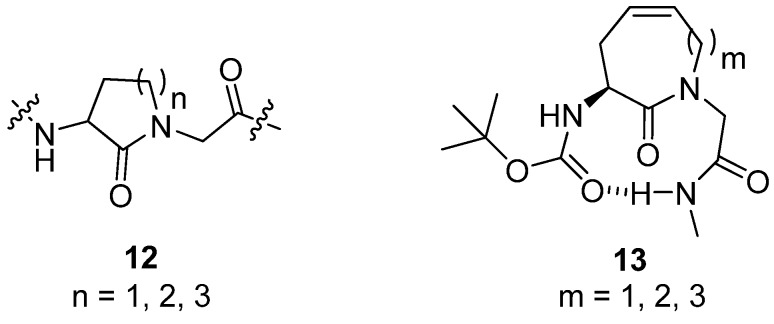
A conformational equilibrium involving a β-turn structure was observed for the dehydro-Freidinger lactam with a 9-membered ring.

Inspired by work by Freidinger on γ- [31], δ- and ε-lactams **12** (Figure 4) the Gmeiner group set out to synthesize dehydro-Freidinger lactams **13** (Figure 4) by RCM and explore their conformational properties, hoping that they might be of value as β-turn mimics [32]. 

It was found that the amount of intramolecular H-bonding between NH^2^ and the carbonyl oxygen of the Boc protecting group increases with increasing ring size when going from a 7-membered ring, via an 8-membered ring, to a 9-membered ring. For the 9-membered ring NOESY experiments revealed the presence of two major conformers. One of the major conformers was found to be a boat-like structure, which is consistent with the presence of a CO(*i*)→NH(*i+3*) H-bond often seen in β-turns. The IR spectrum of the peptide containing a 9-membered ring displays broad peaks at 3,446 cm^-1^ and 3,370 cm^-1^ (more intense), indicative of the presence of a conformational equilibrium involving one intramolecularly H-bonded conformer. 

## 7. C^α^(*i*)→CO(*i+1*), m = 10, H_2_, n = 15 Cyclizations

**Figure 5:**
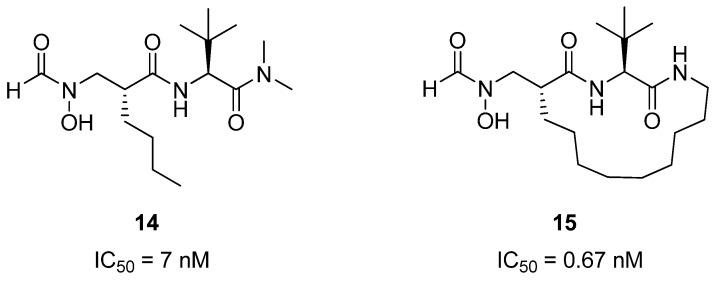
An RCM-derived conformational constraint improves the affinity of **14** for its target peptide deformylase roughly 10-fold.

Peptide deformylase is a eubacterial protein which plays an important role in protein synthesis and maturation, and is a potential drug target for new antibacterial agents [33,34]. Structural studies have shown that peptide inhibitors of PDF related to **14** (Figure 5) bind to the protein in an extended conformation, with the hydrophobic groups on residues *i* and *i+2* buried in a hydrophobic pocket in the PDF active site. Pei and co-workers reasoned that macrocyclization could potentially constrain **14** into the bioactive conformation, thus affording a higher affinity inhibitor of PDF. Indeed, the crosslinked compound **15** is roughly 10-fold more potent than **14 [35,36]**. It also exhibits good antibacterial activity against *Bacillus subtilis* (MIC = 0.7-1.4 μg/mL), but is less active against *E. coli* (MIC = 12 μg/mL *vs*. 8 μg/mL for the acyclic compound). The authors speculate that the lower antibacterial activity may be due to poor membrane permeability, or that the compound is being actively transported out by the bacterial efflux pump. 

## 8. C^α^(*i*)→C^α^(*i+2*), m = 4, (E)/(Z), n = 11 Cyclizations 

**Scheme 1:**
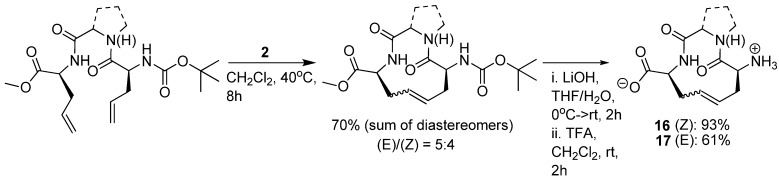
Synthesis of inverse γ-turn analogues by RCM.

The γ-turn is a structural motif sometimes occurring in receptor-peptide complexes. One example is provided by the residues Val802-Asn803-Ala804 of hypoxia-inducing factor-1α (HIF-1α) in the complex with the oxygen sensor and therapeutic target [37] factor inhibiting hypoxia inducible factor-α (FIH), an asparaginyl hydroxylase. There are two subtypes of γ-turns, classic γ-turns and inverse γ-turns, which are related to each other by mirror symmetry. The inverse γ-turn is characterized by dihedral angles of (φ,ψ) = (-79 ± 40°, 69 ± 40°) [38,39]. In a recent study, Claridge and co-workers synthesized conformationally restrained cyclic analogues of the inverse γ-turn motif observed in the crystal structure of the HIF-1α-FIH complex [40] (Scheme 1). Both alkene isomers **16** and **17** were isolated and subjected to extensive conformational analysis by low-temperature (238 K) NMR in d_5_-pyridine, revealing the presence of two major conformers for both diastereomers. Each of the conformers were found to have dihedral angles -83°<φ<-80° and 50°<ψ<79° for residue 2, which is in close agreement with the dihedral angles of Asn803 in the crystal structure of the HIF-1α-FIH complex. After replacing glycine in position 2 with proline only one conformer, with a transannular, intramolecular *i→i+2* H-bond, was observed. 

## 9. C^α^(*i*)→C^α^(*i+2*), m = 6, H_2_, n = 13 Cyclizations

Recently, the synthesis of macrocyclic phosphino dipeptide isostere inhibitors of β-secretase, the enzyme that cleaves the N-terminus of β-amyloid precursor protein (APP), by on-resin, microwave assisted RCM was reported [41]. Unfortunately, as a result of RCM, the IC_50_ values increases slightly (from 12 to 47 nM for the most active isomer) compared to the parent compound. However, the serum half-life is tripled (14.8 min to 43.9 min). While the acyclic precursor is completely degraded after 120 min 20% of the initial concentration is still left of the macrocyclic analogue after 160 min. 

## 10. C^α^(*i*)→C^α^(*i+2*), m = 8, (E)/(Z), n = 15 Cyclizations

**Scheme 2:**
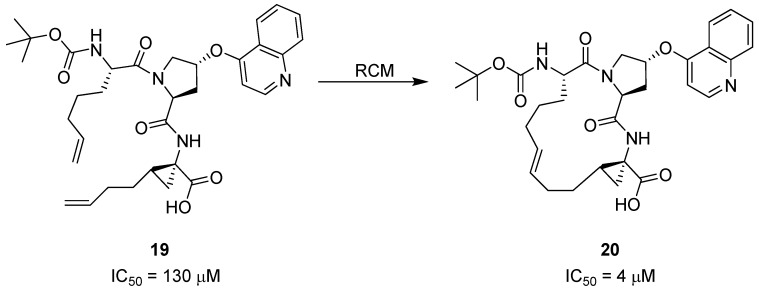
Macrocyclization of the HPC NS3 inhibitor **19** to yield **20** reduces the IC_50_ value 32-fold.

More than 170 million people worldwide are infected by hepatitis C (HPC) virus, which can lead to chronic liver disease. Researchers at Boehringer Ingelheim have identified the hexapeptide Asp-Asp-Ile-Val-Pro-Cys **18** as a weak inhibitor (K_i_ = 79 μM) [42] of the viral NS3 serine protease, which plays an essential role in the viral lifecycle. From NMR studies of the complex between similar hexapeptides and NS3 it was concluded that these peptides bind in an extended, β-strand conformation [43]. Importantly, the three C-terminal residues were found to mediate the most important interactions with the enzyme. This allowed a reduction in ligand complexity from hexapeptides to tripeptides [44]. The shorter peptides, such as **19** (Scheme 2), were also found to adopt a β-strand conformation upon binding. However, the bound conformation, particularly of the 1-aminocyclopropylcarboxylic acid (ACCA) moiety, is different from the solution phase conformation [45]. This led the Boehringer Ingelheim scientists to explore macrocyclization by RCM as a means to preorganize the amide backbone in a β-strand geometry and the ACCA residue in its high-energy conformation – in the hope that this would serve to increase the binding affinity for NS3 by compensating for the entropic penalty associated with refolding of the ligand. 

Indeed, macrocyclization proved to be an efficient strategy, yielding a series of cyclic tripeptide scaffolds, exemplified by **20** (Scheme 2), with 5-40 fold higher affinity than their corresponding open-chain analogues, e.g., **19** [45]. 

**Figure 6:**
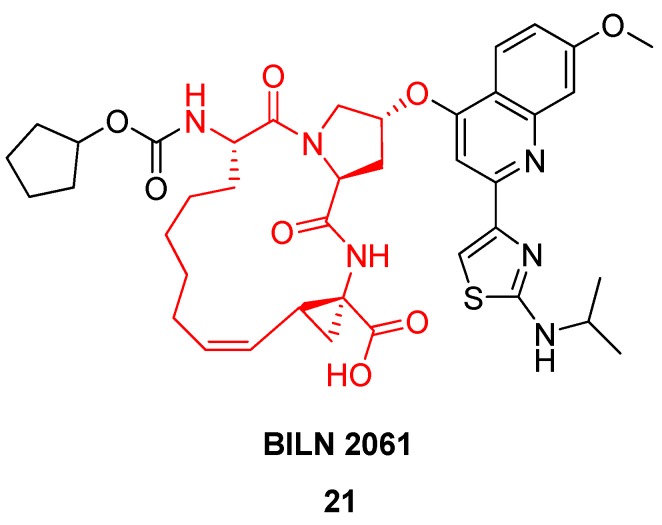
The hepatitis C NS3 protease inhibitor BILN 2061 **21** is based on a 15-membered RCM-derived tripeptide macrocycle (highlighted in red) and showed great promise in phase I clinical trials, but eventually did not make it to the market.

Based on a similar RCM-derived scaffold the Boehringer Ingelheim team developed the antiviral agent BILN 2061 **21** (Figure 6), which made it into clinical trials, where it showed very great promise in phase I [42,46]. In patients receiving 200 mg as an oral solution in a PEG400:ethanol mixture twice daily for 2 days the virus load is reduced to under the detection limit within one day [42]! Unfortunately, the drug candidate never made it to the market due to cardiac toxicity. 

Solution phase NMR studies [45] and the co-crystal structure of a cyclic tripeptide-NS3 complex [47] demonstrated that the solution phase conformations of the cyclic peptides are very similar to the bound conformations, providing proof that ring closure by RCM indeed constrains the ligand in the bioactive conformation. 

As a final note one would think that a process leading to a complicated tricyclic scaffold like that of BILN 2061 **21** would be tough to scale up, but Boehringer Ingelheim scientists managed to produce more than 400 kg of BILN 2061 **21** using the 1st generation Hoveyda (pre)catalyst **5** (Figure 2) in toluene [22]. In an improved process this was replaced by 0.1 mol% Grela’s catalyst **6** (Figure 2) [48]. 

## 11. C^α^(*i*)→C^α^(*i+2*), m = 10, (E)/(Z)/H_2_, n = 17 Cyclizations

**Figure 7:**
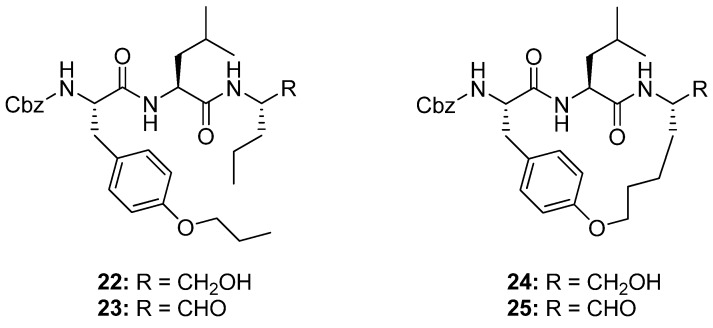
The macrocyclic cystein protease inhibitors **24** and **25** display greater selectivity for the cysteine protease o-CAPN2 than **22** and **23**.

Generally, peptides adopt a β-strand geometry, defined by -160°<φ<-100° and 90°<ψ<160°, when binding to the active site of proteases [49]. This fact has been exploited in the design of macrocyclic β-strand templates for use in the development of inhibitors of cysteine proteases [10,23]. Molecular modeling studies indicate that macrocyclic analogues of **22** and **23** (Figure 7) will be constrained into a β-strand conformation. When assayed for activity against ovine calpain 1 (o-CAPN1) and ovine calpain 2 (o-CAPN2) the macrocyclic compounds **24** and **25** (Figure 7) were found to be more active against o-CAPN2 by a factor of 4 and 22 for the aldehyde **25** (IC_50_ = 30 nM) and alcohol **24** (IC_50_ = 700 nM) respectively. The IC_50_ value for inhibition of o-CAPN1 increases by a factor of 4 for the aldehyde **25** (IC_50_ = 220 nM) and decreases by a factor of 2 for the alcohol **24** (IC_50_ = 1.75 μM) relative to their acyclic analogues [10]. When tested against cathepsin B, a cysteine protease belonging to the same class as calpain, the IC_50_ values were found to be greater for the macrocyclic compounds (IC_50_ = 70 and 300 nM for the aldehyde **25** and alcohol **24** respectively) than for their acyclic analogues **22** and **23** (IC_50_ = 5 and 200 nM for the aldehyde and alcohol respectively). Finally, the cyclic aldehyde **25**, which is the most active compound overall against o-CAPN2, was tested for its ability to retard the development of calcium-induced cortical cataracts. At a p < 0.005 confidence level the compound was shown to significantly reduce lens opacity after 6h incubation of sheep lenses in a 1 μM solution. 

**Scheme 3:**
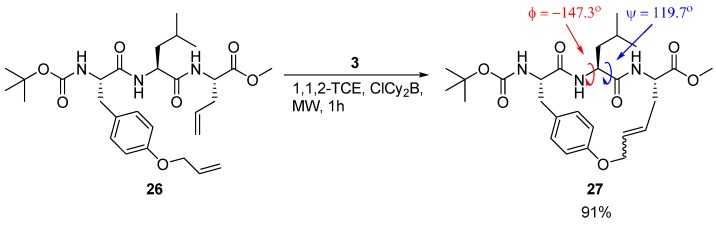
The precursor **27** of the cysteine protease inhibitors **24** and **25** adopts the β-strand geometry required for binding to a protease.

A detailed X-ray crystallographic structural investigation was undertaken for a cyclic olefinic precursor **27** of **24** and **25**, revealing that **27** adopts a β-strand geometry required for binding to a cysteine protease, with (φ,ψ) = (-147.3°, 119.7°) for the Leu residue between the bridgeheads (Scheme 3) [23]. 

**Scheme 4:**
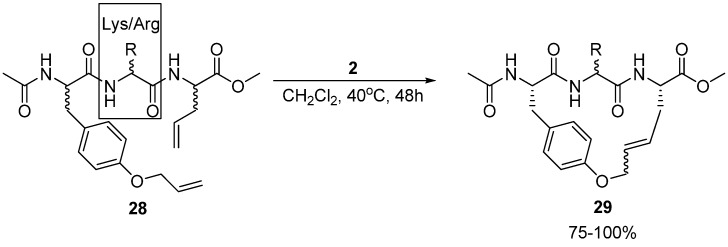
Synthesis of macrocyclic tripeptides **29** with activity against *S. aureus*. Note that the cyclization was performed on a side chain protected variant.

Multiresistant bacteria constitute a growing health problem worldwide. There is therefore an urgent need for new antibacterial agents. One promising avenue of research is short, cationic peptides, whose minimal anti-staphylococcal pharmacophore has been proposed to consist of two units of charge and two units of (hydrophobic) bulk [50]. Boyle and co-workers have synthesized and assayed the antibacterial activity of a series of acyclic and cyclic olefinic tripeptides against *Staphylococcus aureus*, varying the stereochemistry of the three residues and the identity of the positively charged residue in position 2 (Lys or Arg) (Scheme 4) [51]. In all but one case the activities of the cyclic compounds **29** were found to be higher than for their acyclic analogues. The most active cyclic tripeptide with the general structure given in Scheme 4 is the (L,D,L)-isomer, with the positively charged residue being D-arginine. Its minimum inhibitory concentration (MIC) is a modest 62.5 μg/mL. However, the acyclic analogue **28** is inactive (MIC > 125 μg/mL). Surprisingly perhaps, the most active compound identified by Boyle and co-workers is not a tripeptide at all, but a dipeptide intermediate, having a relatively low MIC value of 7.8 μg/mL. For reference, vancomycin has MIC = 1.25-2.5 μg/mL. 

## 12. C^α^(*i*)→C^α^(*i+2*), m = 16, (E)/(Z), n = 23 Cyclizations 

In the study of macrocyclic antibacterial tripeptides mentioned earlier Boyle and co-workers also investigated the effect of substituting the allylglycine residue in **28** (Scheme 4) with *O*-allyl-tyrosine [51]. The antibacterial activity of the resulting acyclic compound is better than that of the allylglycine containing peptide, but is nevertheless quite low (MIC = 125 μg/mL). However, the cyclic compound, with an RCM-derived bridge, is significantly more active (MIC = 15.6 μg/mL). 

## 13. N(*i*)→CO(*i+2*), m = 7, (E)/H_2_, n = 16 Cyclizations 

**Figure 8:**
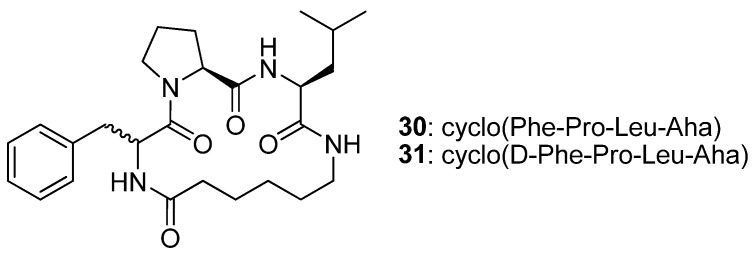
In **30** the Phe-Pro element adopts a type VIa2 β-turn conformation, whereas in **31** the Phe-Pro element folds into a type II’ β-turn conformation.

The cyclic tetrapeptides cyclo(Phe-Pro-Leu-Aha) **30** and its epimer cyclo(D-Phe-Pro-Leu-Aha) **31** (Figure 8) were synthesised from the allylamides of *N^1^*-(4-pentenoyl)-Phe-Pro-Leu-Aha (**32**) and *N^1^*-(4-pentenoyl)-D-Phe-Pro-Leu-Aha (**33**) by RCM followed by catalytic hydrogenation, and their crystal structures solved by single crystal X-ray diffraction [52]. The structure of **30** features a type VIa2 β-turn [(φ,ψ)_Phe_ = (-169.3°, 135.6°), (φ,ψ)_Pro_ = (-75.2°, -19.3°), ideal: -120°, 120° and -60°, 0°] [52,53] around the Phe-Pro residues. The Pro amide bond is in the *cis* configuration and all donor and acceptor groups are roughly perpendicular to the surface defined by the backbone of the peptide, with the result that no intramolecular H-bonds are observed. Replacement of L-Phe with D-Phe results in a different conformation, with two 10-member transannular, intramolecular H-bonds. The conformation of the backbone changes to a type II’ β-turn, with (φ,ψ)_D-Phe_ = (48.8°, -131.5°) and (φ,ψ)_Pro_ = (-69.5°, -10.9°) (ideal: 60°, -120° and -80°, 0°), around D-Phe-Pro, and to a type I β-turn around the Pro-Leu residues [(φ,ψ)_Pro_ = (-69.5°, -10.9°), (φ,ψ)_Leu_ = (-101.1°, 10.2°); ideal: -60°, -30° and -90°, 0°]. This study illustrates the major impact that the stereochemistry of the residues may have on the structure of a cyclic peptide. 

**Scheme 5:**
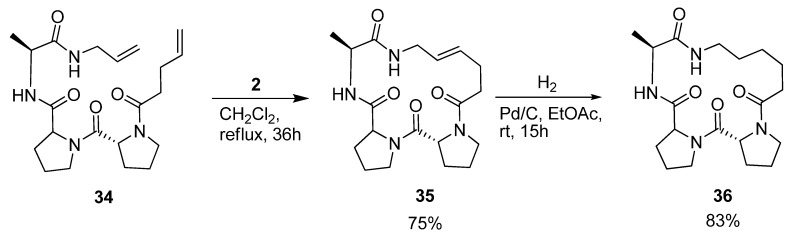
The acyclic tripeptide **34** and its cyclized hydrogenated analogue **36** both adopt a minimal 3_10_ helical conformation in solution according to NMR spectroscopic evidence.

Iqbal and co-workers were interested in the conformational properties of the acyclic peptide **34** and its cyclic variants **35** and **36** (Scheme 5) because of the potential utility of D-Pro-L-Pro templates as starting points in protein ligand, vaccine and receptor antagonist design [54]. NMR spectroscopy indicates the presence of two successive β-turns in the solution structure of **34**, the first, involving pentenoyl-D-Pro-L-Pro-Ala, being of type II’, and the second, involving the D-Pro-L-Pro-Ala-allyl amide, of type I. This indicates that the peptide bears some resemblance to a minimal 3_10_ helix. The presence of a strong minimum at 205 nm and a shoulder at 215 nm in the CD spectrum corroborates the conclusions drawn from the NMR studies. Cyclization by RCM followed by hydrogenation provided **36**, which was characterized by X-ray crystallography. With dihedral angles (φ,ψ) = (52.1°, -138.5°), (-68.5°, -13.4°) and (-74.4°, -17.4°) for the residues D-Pro, L-Pro and Ala respectively, its structure consists of two successive β-turns of the same types as was the case for the acyclic precursor molecule. The dihedral angles of the L-Pro and Ala residues all fall within ±17° of an ideal 3_10_ helix ((φ,ψ) = (-57°, -30°). The crystal structure also reveals two intramolecular *i*→*i+3* H-bonds, which are often present in β-turns. Consecutive intramolecular *i*→*i+3* H-bonds constitute a defining feature of 3_10_ helices. 

**Scheme 6:**
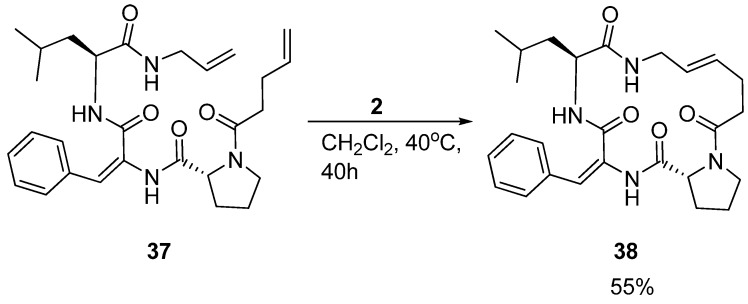
The presence of a dehydrophenylalanine residue changes the structural outcome of RCM in proline containing tripeptides similar to **34**.

Interestingly, in a separate study by the Iqbal group aiming at developing small cyclic β-turn mimics for development of HIV-protease inhibitors, another tripeptide **37**, with different primary structure, featuring a dehydrophenylalanine residue, was also shown to adopt a 3_10_ helical structure in solution. However, the cyclic peptide **38** (Scheme 6) adopts a simple β-turn structure [55]. 

## 14. C^α^(i)→C^α^(i+3), m = 4, (E)/(Z)/H_2_, n = 14 Cyclizations

**Scheme 7:**
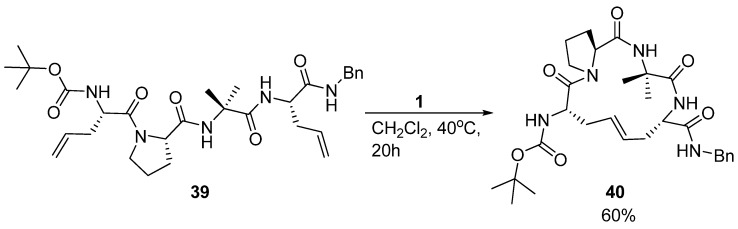
The first reported example of RCM in a peptidic system, replacing a disulfide bond in a β-turn peptide with an olefinic dicarba analogue.

β-turns are important structural elements in peptides and proteins. One type of β-turn is stabilized by an intramolecular H-bond between the carbonyl group of residue *i* and the amide NH of residue *i+3*. Nature stabilizes many β-turn motifs by intramolecular disulfide bridges. Miller and Grubbs, in what was the very first application of RCM in a peptidic system, replaced a disulfide bridge in a β-turn peptide reported by Balaram and co-workers [56] with a dicarba analogue derived from RCM affording **40** (Scheme 7) [4].

The general idea of replacing a disulfide bond with a potentially metabolically more stable alkene [57] had been illustrated much earlier [58], but Miller and Grubbs were the first to use RCM to install the alkene. As the focus was on demonstrating the feasibility of RCM in a peptidic system, no detailed structural information was obtained on how replacement of a disulfide bond, which has different dihedral angle requirements than an olefin (82° *vs*. 0° or 180°) [59], affected the structure. However, in a follow-up study IR spectroscopy, temperature gradient and solvent titration ^1^H-NMR experiments demonstrated that an intramolecular H-bond between residues 1 and 4 in the disulfide parent peptide is preserved in the olefinic analogue **40**, suggesting that the structural perturbation of the backbone is minor [9]. 

The acyclic precursor was prepared as a mixture of four diastereomers by allowing both configurations at the α-carbon atoms of residues 1 and 4. However, only the (*S*,*S*,*S*)-isomer **39** was observed to cyclize readily (in 60% yield) [4]. This is the same stereochemistry as the parent disulfide bridged peptide and suggests that the RCM reaction benefits from a degree of substrate preorganization of the acyclic precursor **39**, possibly due to the intramolecular H-bond.

**Figure 9:**
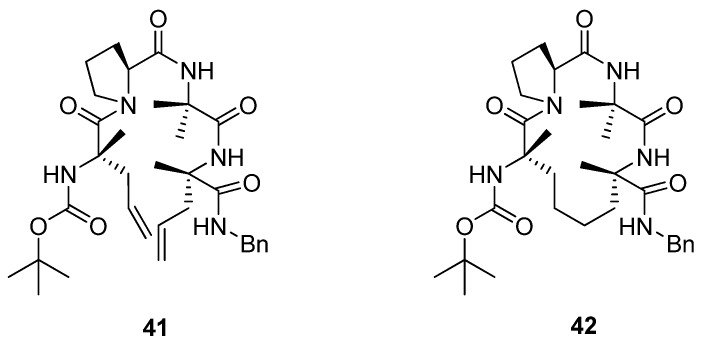
The cyclic peptide **42** and its acyclic precursor **41** both adopt compact type-II β-turn conformations in solution, probably due to the steric requirements of the α,α-disubstituted residues.

Later, Toniolo and co-workers studied a dimethyl-substituted hydrogenated variant **42** of Grubbs’ cyclic peptide **40** [60] (Figure 9). A very strong ROESY cross-peak was observed between the C^α^(2)H and N(3)H protons, typical of a type-II β-turn. Likewise, the open-chain precursor **41** appears to be folded in a compact β-turn conformation. The authors attribute this to the presence of the two α,α-disubstituted C^α^-methyl-L-allylglycine residues, which put severe constraints on what backbone conformations the peptide may adopt.

**Figure 10:**
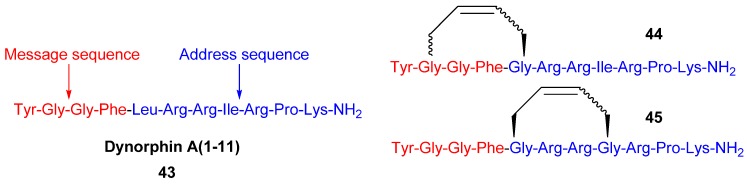
The endogenous analgesic peptide dynorphin A(1-11) **43** and the carbocyclic analogues **44** and **45** synthesized by RCM.

Since the discovery of endogenous analgesic peptides acting at opioid receptors there has been considerable interest in developing peripherally acting κ selective opioid agonist analgesics, which potentially do not have the serious side effects of morphine and other narcotic analgesics [61], which mainly act on μ opioid receptors. Dynorphin-A(1-11) **43** is an analgesic peptide consisting of a “message” sequence (Tyr-Gly-Gly-Phe), which is the same as for most other mammalian opioid peptides, and an “address” sequence that is unique to Dyn A(1-11) [62] (Figure 10). Recently, cyclic analogues **44** and **45** of the κ receptor agonist dynorphin A(1-11), some of which show improved κ receptor selectivity compared with the parent peptide (K_i,κ_/K_i,μ_/K_i,δ_ = 1/3/11), were synthesized by RCM [19] (Figure 10). However, each macrocyclic analogue displays lower affinity for each of the opioid receptor subtypes, including the κ receptor, than the parent peptide. The observed increase in κ receptor selectivity is thus due to a greater reduction in affinity for the μ and δ receptors than for the κ receptor. Whether the macrocycle is installed in the “message” or “address” part of Dyn A(1-11) (Figure 10) is of less significance for the selectivity, but the affinities are higher when installed in the address sequence. In the compounds with the macrocycle in the “message” sequence the stereochemistry of the allylglycine residue in position 2 was observed to have a significant influence on the κ receptor selectivity, with only the (L)-isomer displaying improved selectivity. However, in contrast to the (L)-isomer the (D)-isomer has comparable affinity to the parent peptide. The stereochemistry of the double bond is also of some importance for both the affinity and the κ selectivity. The (*E*)-geometry gives in all but one case the same or higher affinity for all opioid receptor subtypes and, for the 2-(L)-isomers, also better selectivity. All in all, taking both affinity and selectivity into account, the optimal combination appears to be installing the macrocycle with (*E*)-stereochemistry in the address sequence, thus leaving Gly2 unsubstituted.

In an earlier study by the Schiller group dicarba analogues of the structurally similar enkephalin-derived pentapeptides H-Tyr-c[D-Cys-Gly-Phe-D(or L)-Cys]-NH_2_ **46** and **47** with different Cys5 and double bond stereochemistry were synthesized and their opioid activities assayed [63]. In addition, the hydrogenated variants were also tested. All compounds have K_i_ values for binding to μ and δ opioid receptors within the same order of magnitude as the parent disulfide bridged peptides. However, the hydrogenated dicarba-L-Cys5 and olefinic (*Z*)-dicarba-D-Cys5 compounds were observed to have significantly higher (~ 10-fold) selectivity for μ/δ *vs.* κ receptors. To verify that the RCM derived bridge makes the cyclic peptides more stable towards redox enzymes like disulfide reductase the compounds were incubated with rat brain suspensions for 24 h at 37 °C. No degradation of the peptides was observed. 

**Figure 11:**
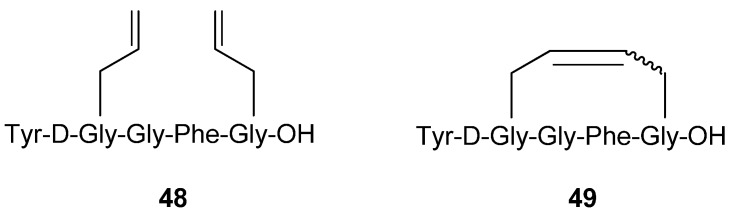
The cyclic (*E*)-olefin **49** was found to be significantly more potent against opioid receptors than the acyclic precursor **48**.

In parallel with the Schiller group the group of Hruby synthesized and evaluated the potencies of completely analogous compounds, with the very slight difference that the terminal primary amide group was substituted with a carboxylic acid functionality [64] (Figure 11). Importantly, Hruby and co-workers also tested the acyclic precursor **48**, which was found to be 20-fold less potent (K_i_ values) against δ receptors and 50-fold less potent against μ and κ receptors than the (*E*)-olefin **49**. All compounds exhibit high selectivity towards the μ/δ receptors, as is common for enkephalin analogues. 

## 15. C^α^(*i*)→C^α^(*i+3*), m = 5, (E)/(Z)/H_2_, n = 15 Cyclizations

**Figure 12:**
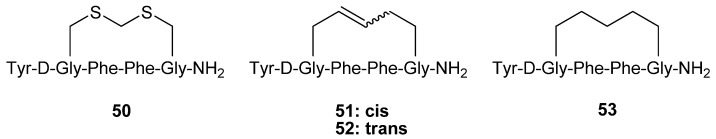
Replacing the dithioether linkage in **50** with a dicarba bridge leads to a reduction in potency and selectivity for opioid receptors.

The hexapeptide **50** (Figure 12) is a highly potent, selective μ opioid receptor agonist (K_i,μ_/K_i,δ_/K_i,κ_ = 1/112/156, K_i,μ_ = 16 pM) cyclized via a methylene dithioether. Schiller and co-workers synthesized the analogues **51-53** by RCM and evaluated their opioid receptor affinities and selectivities [65]. 

The selectivity for the μ receptor over the δ receptor was found to be almost completely abolished, whereas the selectivity for μ over κ is reduced ~ 4-fold. This is accompanied by a roughly 20-fold and 5-fold decrease in the affinity for the μ and κ receptors and a virtually unchanged affinity for the δ receptor respectively. 

## 16. C^α^(*i*)→C^α^(*i+3*), m = 8, (E)/H_2_, n = 18 Cyclizations

**Figure 13:**
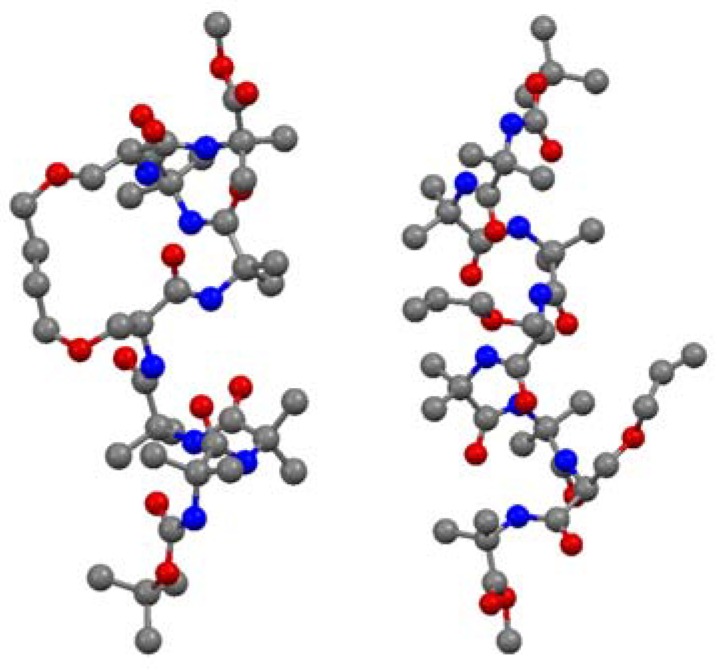
X-ray crystal structures of a 3_10_ helix before (right) and after (left) crosslinking by RCM. Hydrogen atoms have been omitted for clarity.

Short peptides containing multiple α-aminoisobutyric acid (Aib) residues show high propensity for folding into 3_10_ helices. The 3_10_ helix is defined by intramolecular *i→i+3* H-bonds and dihedral angles (φ,ψ) = (-57°, -30°). Theoretical studies by Toniolo and co-workers indicate that a minimal RCM- derived conformational constraint for a 3_10_ helix consists of 8 atoms in a bridge linking the α-carbon atoms of residues *i* and *i+3* [66]. O’Leary and co-workers set out to test this hypothesis in a model peptide which was expected to have a high intrinsic propensity to form a 3_10_ helix due to its high Aib content. Ring-closing using Grubbs’ 2nd generation catalyst **3** proceeds in high (93%) yield and with unusually high (>20:1) (E)/(Z)-selectivity [67]. The X-ray crystal structures of the acyclic precursor *and* the crosslinked product allow strong conclusions to be drawn on the effect of RCM on the backbone structure of the peptide [67] (Figure 13).

The acyclic peptide adopts a 3_10_ helical fold from residue 1 to residue 6. Residue 7 forms, together with residue 6, a type-I β-turn. Most of the dihedral angles of the cyclic compound and its hydrogenated analogue were found to lie within 10° of the corresponding dihedral angles in the acyclic precursor. The largest deviations were observed at the two monosubstituted *O*-allyl-L-serine residues, with [|Δφ|,|Δψ| = 22°, 39° and 14°, 16°]. Apart from an intramolecular H-bond between N6 and O3, which is absent in the cyclic olefinic peptide, all intramolecular H-bonds present in the acyclic peptide are also present in the cyclic compounds. The CD spectra in methanol indicate that the solution phase structures of all compounds are quite similar and consistent with a 3_10_ helix. 

**Figure 14:**
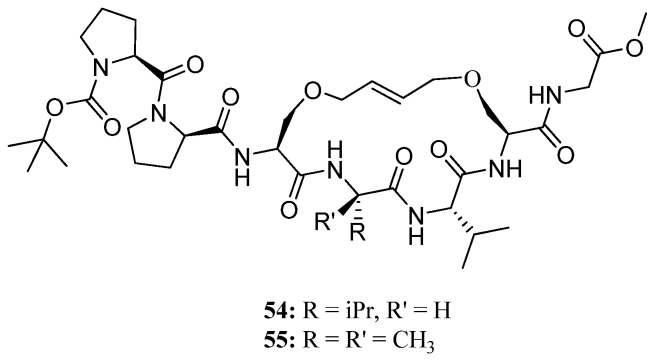
Cyclic analogues **54** and **55** of the Pro138-Gly144 segment of human AQP4 stabilized in a 3_10_ helical conformation by an RCM-derived bridge linking the side chains of residues 3 and 6.

Recently, this methodology was applied to the synthesis of the conformationally constrained cyclic analogues **54** and **55** of the 3_10_ helical Pro138-Gly144 segment of the human water channel aquaporin-4 (AQP4) mediating adhesive interactions between AQP4 tetramers [68] (Figure 14). AQP4 is an important potential target for drugs against brain edema, a common consequence of stroke and trauma [69,70]. NMR and CD data demonstrated that the peptides **54** and **55** can access 3_10_ helical conformations in polar aprotic (CD_2_Cl_2_) solvents and, most importantly, indicated that the olefinic bridge has a significant stabilizing effect on helicity in an aqueous environment. The authors envision developing **54** and **55** into molecular scaffolds with binding affinity for AQP4, which potentially can be elaborated upon to provide inhibitors of AQP4 [68].

## 17. “N”(*i*)→C^α^(*i+3*), m = 3 and 4, (E)/(Z), n = 13 and 14 Cyclizations 

**Figure 15:**
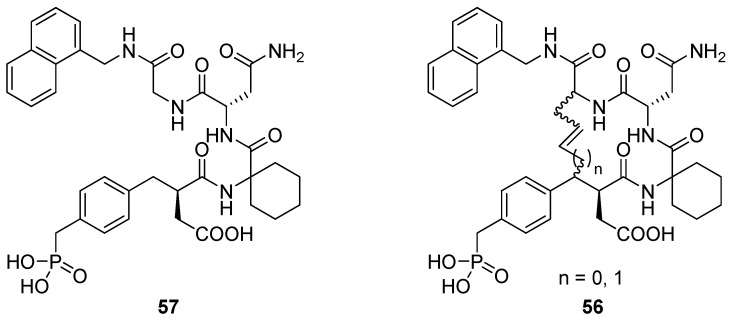
Macrocyclization by RCM converts **57** from a low micromolar to nanomolar Grb2 SH2 domain binding ligands.

Grb2 SH2 domains are recognition motifs involved in a range of cellular signalling pathways and could be therapeutically important drug targets [71]. These protein domains primarily recognize sequences of the type pTyr-Xaa_1_-Asn-Xaa_2_ in type-I β-turns. Burke and co-workers utilized RCM to prepare a series of macrocyclic analogues **56** of the low micromolar (K_D_ = 5.6 μM) Grb SH2 domain-binding ligand **57** (Figure 15) with varying bridge lengths (m = 3 or 4), positioning and stereochemistry of the resulting double bond and stereochemistry at the bridgeheads [72,73,74]. Formally, these cyclizations could be classified as N(*i*)→C^α^(*i+2*) cyclizations, but the close similarity between the N-terminal part of **57** and a substituted aspartic acid residue makes it more natural to classify them as “N”(*i*)→C^α^(*i+3*) cyclizations. Each of the macrocyclic ligands were found to have dissociation constants in the range 20-70 nM, *i.e.* approximately 100-fold lower than the acyclic parent compound, thus providing a nice example of the positive effect macrocyclization by RCM can have on the affinity of a peptidic ligand. It should be added that the magnitude of the observed affinity enhancements was slightly dependent on whether the K_D_ values were determined under steady-state conditions or calculated from association and dissociation rate constants [74]. In the latter case they were “only” on the order of 10-30-fold lower than for the acyclic compound. 

In solution phase NMR temperature coefficients suggest that two of the three NHs in the macrocycle are solvent shielded and thus could be involved in intramolecular H-bonds. Further evidence for intramolecular H-bonding is provided by deuterium exchange experiments, in which the endocyclic NHs are found to exchange slower than the exocyclic amide groups. In particular, for the acyclic compound deuteration is slowest for the amide group of the glycine residue. This is exactly the NH group which would be expected to be involved in an *i*→*i+3* transannular H-bond if the peptide adopts a β-turn conformation. 

## 18. N(*i*)→C^α^(*i+3*), m = 13, (E), n = 24 Cyclizations

**Figure 16:**
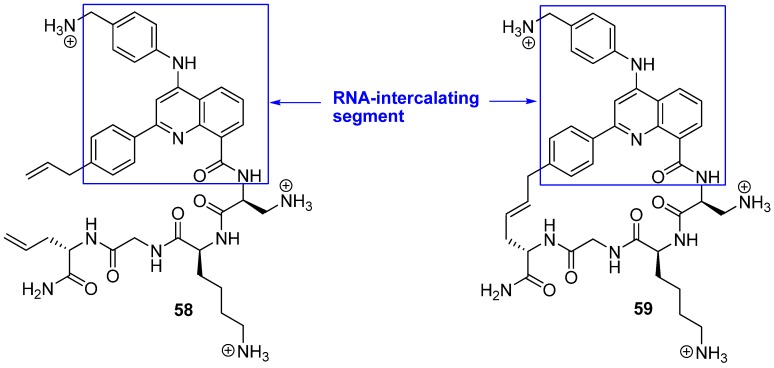
Macrocyclization of the helix-threading peptide **58** affords a higher affinity ligand.

While almost all efforts to design conformationally constrained peptides have been directed towards obtaining better or more stable ligands for protein targets the work by Beal and co-workers [75] differs in two respects from the norm. Firstly, RNA is the target of their peptides. Secondly, the bridge is part of the motif interacting most strongly with the target. Peptides **58** and **59** belong to a class of helix-threading peptides (HTPs) which contain a heterocyclic core that intercalates certain RNAs (Figure 16). The dissociation constants for the complexes between the cyclized compound and two different RNAs known to have a high-affinity HTP binding site were measured to be 6-7 fold smaller than for the complexes containing the acyclic analogue [75].

## 19. N(*i*)→CO(*i+3*), m = 7, (E)/(Z)/H_2_, n = 19 Cyclizations 

**Scheme 8:**
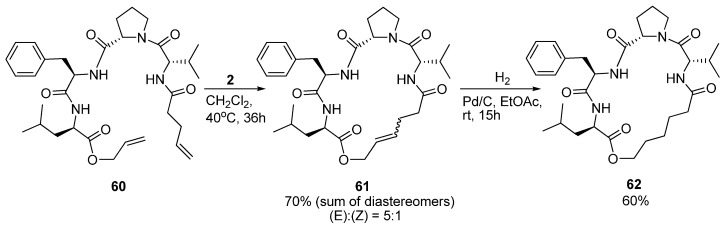
The saturated macrocyclic tetrapeptide **62** adopts a type VIa β-turn structure in CDCl_3_ and a type VIb β-turn structure in the more polar solvent DMSO-d_6_.

As part of a study on the development of small cyclic peptide inhibitors of proteases Iqbal and co-workers observed that an *N*-cinnamoyl methyl ester analogue of **60** (Scheme 8) exists in a 3_10_ helical conformation in solution. In order to set up the system for RCM the diolefin **60** was prepared. Surprisingly, in contrast to its *N*-cinnamoyl methyl ester analogue, **60** was observed not to be 3_10_ helical anymore [76]. NMR spectroscopy indicated the presence of multiple structures. At first, cyclization to **61** did not appear to stabilize the peptide in any particular conformation. However, upon removal of the double bond by catalytic hydrogenation (Scheme 8) detailed ^1^H-NMR studies revealed the presence of a type VI β-turn motif [76]. The conformation of **62** was found to be slightly solvent-dependent, with a type VIa β-turn being favoured in CDCl_3_ and a type VIb β-turn in DMSO-d_6_. To corroborate their findings Iqbal and co-workers also recorded the CD spectrum and found that its shape matches the spectra previously reported for type VI β-turns [77].

## 20. N(*i*)→N(*i+3*), m = 7 and 8, (E)/(Z), n = 17 and 18 Cyclizations

**Scheme 9:**
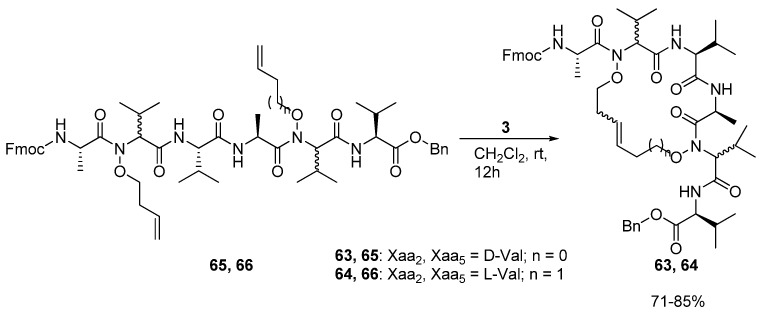
Cyclization of the *N,N’*-dialkenoxy peptides **65** and **66** proceeds in good yield, but the resulting cyclic peptides **63** and **64** appear to be unstructured in solution.

In 2008 Blandin and co-workers reported the synthesis of the cyclic peptides **63** and **64**, which are derived from the *N,N’*-dialkenoxy peptides **65** and **66** [78] (Scheme 9). 

The alkenoxy groups were introduced by DMAD/PPh_3_ mediated coupling of *N,N’*-dihydroxy peptides with unsaturated alcohols. Ring closure is facile and proceeds in 71-85% yield. However, in contrast to the products of analogous C^α^(*i*)→C^α^(*i+3*) cyclizations, the peptides do not adopt any particular secondary structure according to preliminary structural investigations by NMR spectroscopy and restrained molecular dynamics. 

## 21. C^β^(*i*)→C^β^(*i+3*), m = 8, (E)/H_2_, n = 21 Cyclizations

**Scheme 10:**
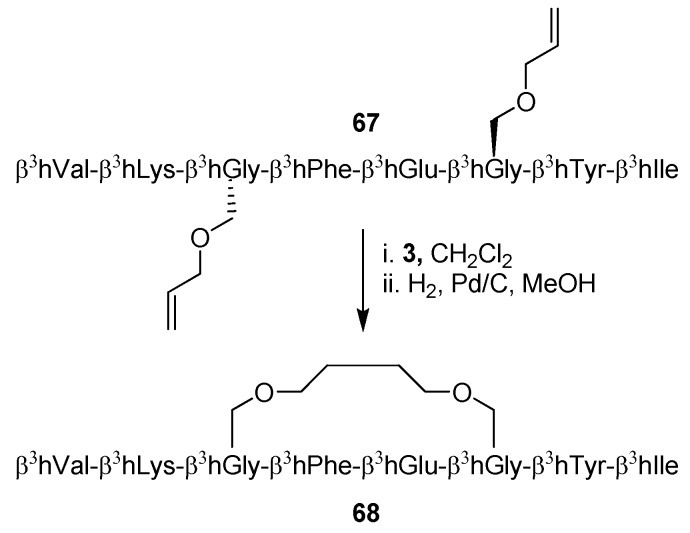
RCM applied to a β^3^-octapeptide.

Whereas RCM has been extensively applied to α-peptides it is only recently that the first examples of RCM applied to β-peptides have appeared [12,13]. Perlmutter and co-workers examined four hexa-β^3^-peptides before and after cyclization and found by NOESY experiments and CD spectroscopy that their structures are consistent with a 3_14_-helix under a range of solvent conditions [12]. A more thorough analysis of the effect of an RCM-derived staple on the structure of β^3^-peptides was offered by Seebach and co-workers, who solved the full NMR structure of the β^3^-octapeptide **67** before and after ring-closing [13] (Scheme 10). Surprisingly perhaps, the conclusions derived from NMR spectroscopy do not fit well with the apparent conclusions from CD spectroscopic investigations, which indicate that the population of 3_14_-helical conformations is much greater (threefold) for the stapled peptide **68** than for the acyclic precursor **67**. In contrast, NMR shows no signs of a greater propensity for the cyclic peptide to fold into a 3_14_ helical structure. In the field of β^3^-peptides the intensity of the negative Cotton effect at ~215 nm is taken as a measure of 3_14_-helical “content”. However, as pointed out by Seebach and co-workers, the CD signal is mainly the result of local electronic structure and conformation, and may thus in certain cases be misleading when drawing conclusions about the global structure of a peptide [13].

## 22. C^α^(*i*)→C^α^(*i+4*), m = 6, (E)/(Z)/H_2_, n = 19 Cyclizations

In a study which represents an interesting twist to the use of RCM to constrain peptides into defined shapes Weigand and co-workers prepared cyclic analogues of the K_171_APREKYWL_179_ segment of the membrane-proximal extracellular region of the human high affinity IgE receptor α-chain (ecFcεRIα), which mediates binding of the monoclonal antibody 5H5F8 [79]. Upon binding to ecFcεRIα the antibody 5H5F8 blocks IgE-mediated activation of human basophils/an allergic reaction. Their objective was to make an artificial, simplified IgE receptor that could potentially be used in a screening effort to identify small molecules with the same anti-allergic effect as 5H5F8. The crystal structure of a complex between the peptide KAPREKYWL **69** and the Fab fragment of 5H5F8 revealed that Arg174, Glu175 and Tyr177 mediate the binding to the antigen and that overall the peptide is relatively irregularly folded. However, Arg174 and Tyr 177 adopt an α-helical conformation and the remaining residues an extended or polyproline II (P_II_) conformation (not recognized as such in their paper).

**Figure 17:**
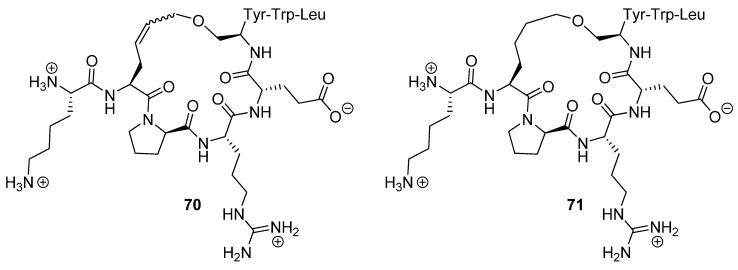
Artificial human high affinity IgE receptor α-chain (ecFcεRIα) receptors for binding of the antibody 5H5F8.

Cyclization was effected by Grubbs’ 1st generation catalyst **2** in CH_2_Cl_2_. Using surface plasmon resonance (Biacore) the dissociation constants of the cyclic peptide **70** and its hydrogenated analogue **71** (Figure 17) were determined to be 5.56 and 1.10 nM respectively. The dissociation constant of the acyclic parent peptide was found to be 3.94 nM, meaning that cyclization does not abolish binding affinity, but does not significantly improve it either. 

## 23. C^α^(*i*)→C^α^(*i+4*), m = 8, (Z), n = 21 Cyclizations 

**Figure 18:**
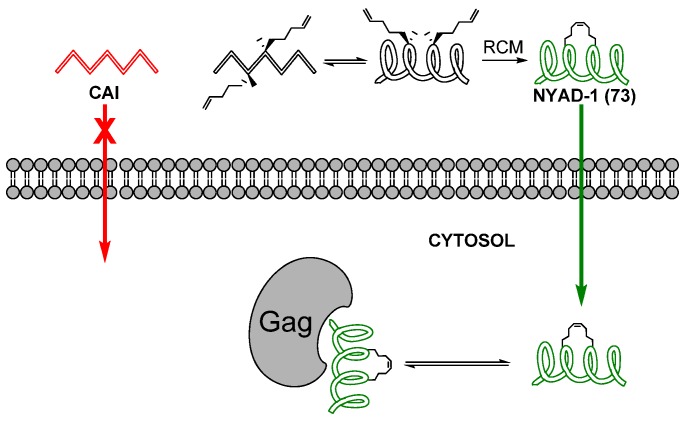
Stapling improves the cell permeability, affinity for the C-CA domain of Gag and anti-HIV-1 activity of the dodecapeptide CAI.

The dodecapeptide CAI (Ile-Thr-Phe-Glu-Asp-Leu-Leu-Asp-Tyr-Tyr-Gly-Pro-NH_2_) **72** (Figure 18) inhibits assembly of both immature- and mature-like HIV-1 virions *in vitro* by binding to the C-terminal domain of capsid (C-CA). Unfortunately, CAI is incapable of inhibiting HIV-1 in cell culture, probably due to its poor cell permeability. Applying the methodology of Walensky and co-workers [80] Debnath and co-workers installed a hydrocarbon staple between residues 4 and 8 by RCM hoping that this modification would render the peptide more cell-permeable [81]. Gratifyingly, the stapled peptide **73**, named NYAD-1, was indeed found to be much more cell-permeable than its acyclic analogue. NYAD-1 conjugated to a fluorescent probe (FITC) stains 96% of all 293T cells and 92% of all MT-2 cells positive, compared to 40% and 0% for CAI conjugated to FITC. The modified peptide was found to co-localize with Gag polyprotein, of which CA constitutes a subdomain after maturation, and to inhibit assembly and maturation of viral particles. Its anti-HIV-1 activity, as measured by inhibition of p24 production in MT-2 cells, was found to be in the low micromolar (IC_50_ ~ 4-15 μM) range for a wide variety of HIV-1-isolates, including AZT-resistant strains. In addition to a much improved cell permeability stapling also renders NYAD-1 a better ligand for C-CA, reducing the dissociation constant from ~ 15 μM for CAI to < 1 μM for NYAD-1. X-ray crystallographic studies revealed that CAI adopts an α-helical conformation when bound to C-CA [82]. However, according to solution-phase structural investigations by CD spectroscopy CAI is unstructured in solution [81]. In contrast to CAI, the stapled peptide is approximately 80% α-helical in solution, suggesting that the staple stabilizes the α-helical conformation and that this preorganization is one of the major reasons for the improved affinity for C-CA [81]. 

## 24. C^α^(*i*)→C^α^(*i+4*), m = 8 and 10, (E)/(Z)/H_2_, n = 21 and 23 Cyclizations

**Figure 19:**
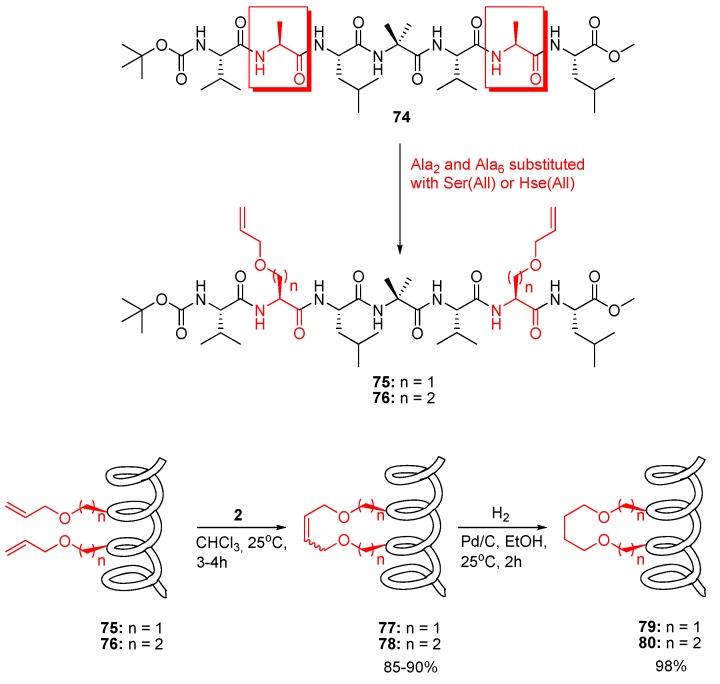
The first application of RCM to predominantly helical peptides.

The hydrophobic peptide **74** studied by Karle and Balaram is known to be α-helical both in CDCl_3_ solution and in the solid state [83] (Figure 19). In a now classic work Blackwell and Grubbs substituted the two Ala residues in Karle and Balaram’s peptide with *O*-allyl-L-serine or *O*-allyl-L-homoserine residues respectively and subjected the resulting diolefinic peptides **75** and **76** to RCM conditions [7,8]. According to CD and NMR spectroscopic evidence all peptides are 3_10_ helical in CDCl_3_ and in the strongly helix-promoting solvent 2,2,2-trifluoroethanol. This work thus constituted the first example of RCM in a pronouncedly helical peptide. The cyclic compound **80** was crystallized and its structure determined by X-ray diffraction. The average dihedral angles of the first five residues are (φ,ψ) = (-57°, -34°), in close agreement with the values characterizing an ideal 3_10_ helix (-57°,-30°) [84]. In addition, the crystal structure features four consecutive *i*→*i+3* intramolecular H-bonds, the defining feature of 3_10_ helices. The presence of H-bonded amide groups in solution phase was confirmed by IR spectroscopy. Finally, solution of the full NMR structure indicated that the deviations from ideal (φ,ψ)-angles are greater for the acyclic peptide **76** than for the cyclic, suggesting that the RCM-derived tether stabilizes the 3_10_ helical conformation [8]. Solvent titration experiments ruled out, however, that the conformational constraint is tight enough to prevent unfolding in a strongly H-bonding solvent, e.g., DMSO [8].

**Figure 20:**
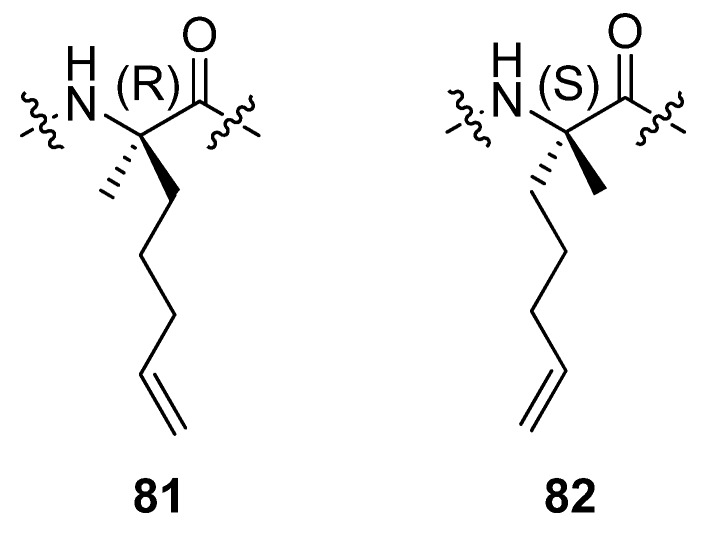
Incorporation of the amino acids **81** and/or **82** at positions *i* and *i+4* in the C-peptide sequence of RNAse A followed by RCM does not result in macrocyclic peptides with enhanced helicity.

In a separate study by the group of Verdine at Harvard incorporation of the α,α-disubstituted amino acids **81** and **82** (Figure 20) in the C-peptide sequence of RNAse A, a peptide known to be only partially helical in aqueous solution, followed by RCM resulted in peptides which are neither more nor less α-helical than the parent peptide [85]. RCM substrates corresponding to bridges with m < 8 do not undergo RCM to an appreciable extent. 

However, the all-hydrocarbon tethering methodology of Verdine [85] was successfully applied to the conformational stabilization of a peptide derived from the BH3 domain of BID, a pro-apoptotic protein that can trigger activation of the pro-apoptotic proteins BAX and BAK, resulting in cytochrome c release and a mitochondrial program of cell death [80]. The binding conformation of the BH3 domain is known to be α-helical. The 23-mer peptide EDIIRNIARHLAQVGDSN_L_DRSIW (N_L_ = L-norleucine) **83** derived from the BH3 domain is merely 16% helical in solution. However, hydrocarbon stapling (m = 8), made possible by substituting Gln13 and Ser17 with **82**, increases this to 35-87%. This results in a more than 6-fold increase in binding affinity (K_d_ = 38.8 nM *vs*. 269 nM for the acyclic peptide) and higher proteolytic stability (t_1/2_ = 29.4 hours *vs*. 3.1 hours in serum *ex vivo*). Most importantly, however, the staple makes the peptide much more cell permeable. One could be tempted to believe that the improved cell permeability is merely due to increased lipophilicity. However, evidence points in the direction of an energy-dependent endocytic process [80]. Further studies established the low micromolar cytostatic effect of a hydrocarbon stapled analogue of the BH3 domain of BID against a wide panel of leukemia cells. Finally, intravenous injection in immunodeficient mice bearing established human leukemia xenografts improves the median survival time after a 300 cGy total body radiation dose from 5 days to 11 days [80]. It was later shown that the stapled peptide exerts its effect by directly binding to and activating the pro-apoptotic multidomain protein BAX [86]. 

In a further, exciting study the *i*→*i+4* stapling methodology was applied to the conformational stabilization of a fragment of MAML1 which has been shown to inhibit NOTCH signalling and cell proliferation of T-cell acute lymphoblastic leukaemia (T-ALL) cells. The stapled peptides exhibit improved α-helicity, cell permeability and, most importantly, curb leukaemic progression in a murine model of T-ALL [87]. 

## 25. N(*i*)→C^α^(*i+4*), m = 6, (E)/H_2_, n = 20 Cyclizations

The SH2 domain of the Grb2 adapter protein, which is a key component of the Ras signalling pathway playing an important role in cell growth and differentiation, has been identified as a potential cancer drug target [88]. 

**Figure 21:**
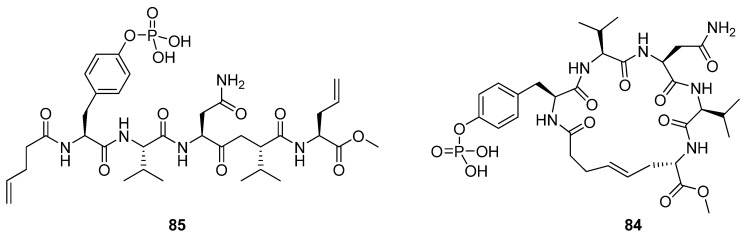
Cyclization of **85** does not improve the affinity for the SH2 domain of the Grb2 adapter protein, possibly because of an enthalpy-entropy compensation effect.

Liskamp and co-workers prepared the cyclic phosphopentapeptide **84** and its hydrogenated analogue (Figure 21), containing the sequence pTyr-Val-Asn-Val, which is known to bind to the Grb2 SH2 domain in a β-turn conformation [89], and compared their affinities with that of the acyclic analogue **85 [15]**. Despite the fact that the modelled minimum energy conformations of the cyclic molecules in the presence of the target domain are very similar to the conformation of the linear pTyr-Val-Asn-Val motif in the co-crystal structure of a linear heptapeptide and the Grb2 SH2 domain, no improvement of the affinity is achieved (K_d_ = 0.60 and 0.48 nM *vs*. 0.44 nM for the linear precursor). The authors speculate that a possible cause for the lack of effect on affinity may be enthalpy-entropy compensation. Sometimes favourable changes in ΔS° can be offset by unfavourable changes in ΔH°, resulting in a vanishing ΔΔG°. However, how the change in ΔS° due to rigidification is counteracted by enthalpy effects remains uncertain. 

## 26. CO(*i*)→N(*i+4*), m = 2, (E)/(Z)/H_2_, n = 13 Cyclizations

A prominent approach to the problem of stabilizing α-helices is to replace the first intramolecular H-bond by a covalent link, forming a macrocycle with the same number of atoms as the parent H-bonded system. According to helix-coil transition theory short α-helices are intrinsically unstable due to low helix nucleation probability. The idea is that by covalently constraining the backbone atoms of the first residues into an α-helical conformation the nucleation barrier is overcome and helix formation initiated. In 1999 Cabezas and Satterthwait pioneered this strategy by replacing the first intramolecular H-bond in an N-acetylated decapeptide with a hydrazone linker between the carbonyl carbon of the N-terminal acetyl group (“residue 1”) and the nitrogen atom of residue 5 [90]. Arora and co-workers have in a series of papers developed this strategy further by replacing the hydrazone link with a more stable hydrocarbon link derived from RCM [91,92,93,94]. A couple of reviews of the literature on so-called hydrogen bond surrogate (HBS) helices have been published recently [95,96]. 

**Figure 22:**
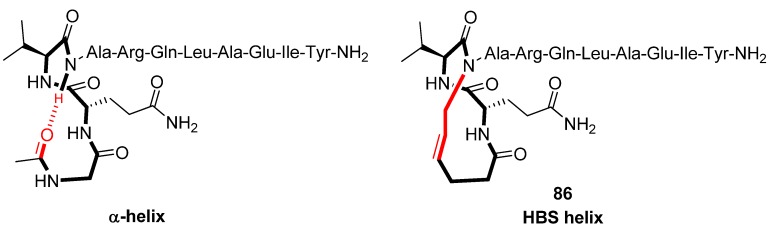
Replacement of an intramolecular H-bond in an α-helix by an RCM-derived covalent link.

Structural studies by CD spectroscopy [92], NMR spectroscopy (amide NH temperature coefficients and deuterium exchange rates) [92,94] and X-ray diffraction (Note: with Ala3 substituted with D-Ala) [93] offer compelling evidence that the HBS helix **86** (Figure 22), derived from the α-helical BH3 domain of the Bak protein, is a highly regular α-helix and thermally more stable than its acyclic analogue. In particular, in 10% 2,2,2-TFE **86** was found to be ~70% α-helical, whereas its acyclic analogue appears to be essentially unstructured [92]. Thermal denaturation studies demonstrated that **86** retains 60-70% of its room-temperature α-helicity even at 85 °C. HBS helices are, however, denatured by concentrated guanidinium chloride [91]. The ideal α-helix is characterized by dihedral angles of (φ,ψ) = (-60°,-42°). In the crystal state the dihedral angles of all residues of **86**, except Gln1 [(φ,ψ) = (113°,-46°)] and the N-terminal residues Ile9 [(φ,ψ) = (-91°,-55°)] and Tyr10 [(φ,ψ) = (-110°,-24°)], fall within the α-helical region of the Ramachandran plot [93].

To evaluate the biological potential of the strategy and to allow a comparison with the side chain cyclization methodology Arora’s group prepared an HBS helix which represents a constrained analogue of a hexadecapeptide derived from the Bak BH3 domain [97]. The Bak BH3 domain is an α-helical domain through which Bak interacts with Bcl-xL, an antiapoptotic protein regulating cell death. The binding affinity of the HBS helix was, however, found to be twofold lower than its unconstrained analogue (325 *vs*. 154 nM). Gratifyingly, the greater helical content (46% *vs*. 20%) results in greater protease stability. Trypsin digestion is roughly 30-fold slower for the cyclic compared to the acyclic compound.

The proponents of the HBS strategy argue that one of the advantages over side chain-to-side chain crosslinking is that burying the stabilizing covalent link in the interior of the helix avoids interference with crucial peptide-protein recognition interactions. Indeed, a side chain lactam crosslinked analogue, prepared by Huang and co-workers, is unable to bind to Bcl-xL [98]. 

The C-terminal transactivation domain (C-TAD_786-826_) of hypoxia-inducible factor 1α (HIF-1α) features two α-helical regions which mediate binding to the CH1 region of the two proteins CBP and p300, thereby initiating transcription of hypoxia-inducible genes, e.g., genes encoding vascular endothelial growth factor (VEGF) and its receptor VEGFR2, which play important roles in cancer growth (angiogenesis) and metastasis. Unfortunately, despite exhibiting significantly greater α-helicity (40-53% *vs*. 15%), HBS analogues of a decapeptide derived from C-TAD-HIF-1α were found to have virtually the same affinity (K_D_ = 420 and 950 nM *vs*. 825 nM) for p300 as the unconstrained analogue [99].

In a further application of the HBS helix methodology the binding affinity of a series of HBS helices for the membrane-anchored gp41 protein – important for HIV-1 membrane fusion – was assayed [100]. Again, the HBS helices do not display appreciably higher affinity for the target than their acyclic analogues (46.6 μM for an HBS analogue of a tetradecapeptide, 37.4 μM for the tetradecapeptide itself; < 5.00 μM for a linear 17-mer and its HBS analogue). On the positive side the 17-mer HBS helix was the only compound found to inhibit gp41-mediated cell fusion [100].

## 27. C^α^(*i*)→C^α^(*i+5*), m = 4, (E)/(Z)/H_2_, n = 20 Cyclizations

Octreotide is a cyclic peptide, containing an intramolecular disulfide bond between residue 2 and 7 (*i.e.* an *i→i+5* disulfide bridge), with high affinity and specificity towards the somatostatin-2 (sst_2_) receptor, a G-protein coupled receptor overexpressed in tumour tissue. The serum half-life is 10 and 22 min when administered by intravenous and subcutaneous injection respectively [101]. It has been shown that replacing the disulfide bridge with an RCM-derived olefinic bridge of the same length and with (Z)-stereochemistry results in a compound that retains a relatively high affinity for the sst_2_ receptor and is stable in serum for more than 30 h [102]. 

Structural studies by NMR spectroscopy indicate that the conformational behaviour of the dicarba analogue is very similar to the parent compound [103]. Like octreotide, the presence of a conformational equilibrium between an antiparallel β-pleated sheet with a type II’ β-turn at the D-Trp-Lys segment and a helical structure is assumed for the dicarba analogue. Despite the apparent structural similarities the binding affinity towards sst_2_ is reduced 20-fold. Towards the other somatostatin receptors sst_5_ and sst_3_ the binding affinities are reduced fivefold and abolished respectively. 

Interestingly, in a recent study it was observed that conjugates of the olefinic dicarba analogue or the corresponding hydrogenated compound with a cytotoxic platinum(II) compound analogous to the famous anti-cancer drug cisplatin, show much improved aqueous stability relative to octreotide-Pt(II) conjugates. In addition, the conjugates bind to DNA in the same fashion as cisplatin [18], thus paving the way for their possible use as targeted, less toxic anticancer agents.

**Figure 23:**
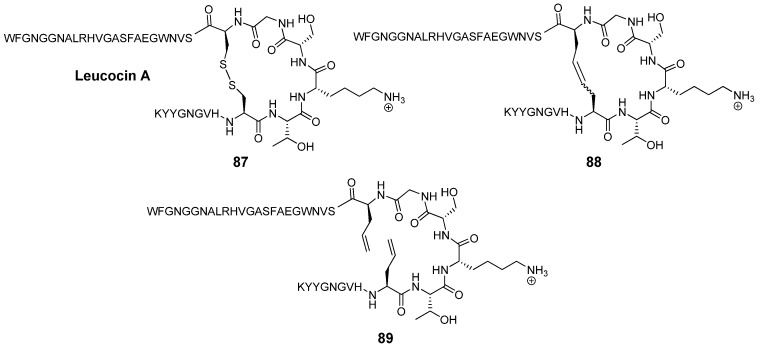
Surprisingly, the diolefinic precursor **89** is nearly as potent against *C. maltaromaticum* as leucocin A **87**. The cyclic dicarba analogue **88** is an order of magnitude less active.

Leucocin A (Leu A) is a naturally occurring antimicrobial 37-mer peptide **87** (Figure 23) with nanomolar activity against *Listeria monocytogenes* and other pathogens [104]. Two disulfide bridged cysteine residues at positions 9 and 14 have been shown to be required for biological activity. The mutations Cys9Ser and Cys14Ser in the highly sequence-similar bacteriocin mesentericin Y105 completely abolish antimicrobial activity. Vederas’ group prepared dicarba analogues **88** of Leu A by RCM and found that, in contrast to the acyclic mutants of mesentericin Y105, they are active against *C. maltaromaticum*, albeit roughly an order of magnitude less so than Leu A (IC_50_ = 370 nM *vs.* 35 nM for Leu A) [105]. Surprisingly perhaps, the diolefin precursor **89** is nearly as active as Leu A (IC_50_ = 50 nM). The authors speculate that in this particular peptide hydrophobic interactions between the two allyl groups are sufficient to stabilize the peptide backbone in the biologically active conformation. Without the strict dihedral angle constraints of the double bond the system can adopt a conformation which closely mimics that of Leu A. Single substitutions of a Cys residue with a hydrophobic residue in the highly sequence-similar bacteriocin pedicocin PA1 abolish antimicrobial activity [106]. 

**Scheme 11:**
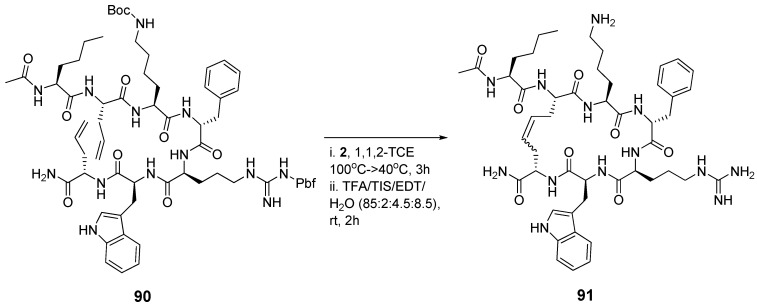
Cyclization of the acyclic MC4-R ligand **90** improves the *in vivo* activity, but not the *in vitro* activity.

The melanocortin-4-receptor (MC4-R) is a potentially important drug target for the treatment of obesity [107]. In an effort to develop novel selective peptide ligands for the MC4 receptor Liskamp and co-workers synthesized the two peptides **90** and **91** (Scheme 11), whose amino acid sequences are derived from the endogenous ligand α-melanocyte stimulating hormone (α-MSH), and measured their affinities towards MC4-R [108]. To their surprise the K_i_ values are very similar (1.9 nM *vs*. 2.3 nM). The EC_50_ values for activation of melanocortin receptors using a gene reporter assay are also virtually identical (0.21 nM *vs*. 0.34 nM for the MC4 receptor), as are their selectivities for MC4-R over MC3-R and MC5-R. A difference in favour of the cyclic compound **91** is, however, found with respect to their *in vivo* abilities to induce excessive grooming behaviour, indicative of MC4 activation, in rats when the compounds are adminstered intracerebroventricularly. The authors speculate that the difference in *in vivo* activity could be due to greater proteolytic stability.

**Figure 24:**
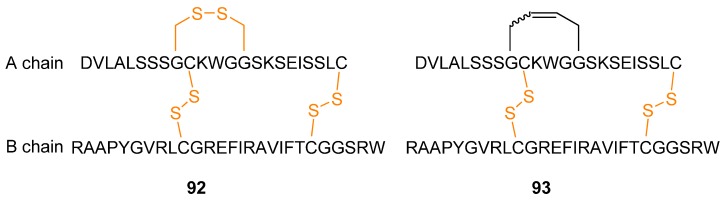
The dicarba analogue **93** of human relaxin-3 **92**, also known as insulin-like peptide 7, retains the solution phase structure and biological activity of the parent polypeptide, with potentially improved *in vivo* stability.

While most applications of RCM to peptides have been directed towards modifying small peptides a notable exception is provided by the work by Wade and co-workers [109]. The polypeptide/small protein human relaxin-3 **92** (Figure 24), an insulin-like peptide which is also known as insulin-like peptide 7 (INSL7), is involved in neurological stress responses and effects on food intake, thus making it a potential drug for treatment of obesity and stress [110,111]. Aiming to develop potentially more stable analogues of human relaxin-3 (e.g., less sensitive to disulfide reductase), a disulfide bridge was replaced with an RCM-derived bridge (Figure 24). 

Structural investigations by CD and NMR spectroscopy revealed that the structure of the protein **93** is virtually unchanged relative to **92** (the α-helix content changes from 36.5% in the native protein to 33-37.2% in the dicarba analogues as judged from the CD ellipticity at 222 nm, [θ_222_]). Biological testing confirmed that the potentially more stable dicarba analogue retains the same biological activity as the native protein with respect to ability to bind to and activate the relaxin-3 receptor RXFP3 and the relaxin receptor RXFP1.

## 28. C^α^(*i*)→C^α^(*i+7*), m = 8-12, (E)/(Z)/H_2_, n = 30-34 Cyclizations

**Figure 25:**
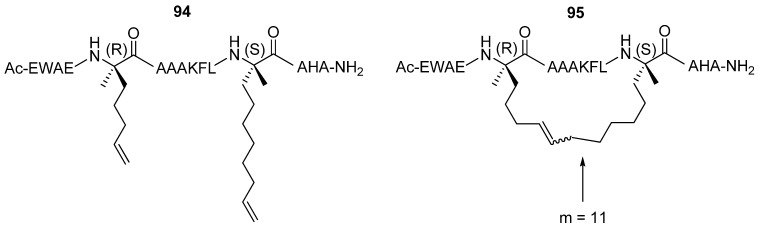
An 11-atom long all-hydrocarbon bridge between residues *i* and *i+7* significantly stabilizes the stapled peptide **95** in an α-helical conformation.

Verdine and co-workers have studied extensively the effect of hydrocarbon stapling, *i.e.* the introduction of an all-hydrocarbon side chain-to-side chain tether by RCM, on α-helicity, binding affinity, cell permeability and other chemical and biological properties for a range of different peptides. After incorporation of α,α-disubstituted amino acids of the type given in Figure 20 the C-peptide sequence from RNAse A **94** was crosslinked by RCM affording stapled peptides **95** with varying bridge lengths [85] (Figure 25). The effect on the α-helicity of the peptide, as measured by CD spectroscopy, was found to be highly dependent on the length of the tether. Tethers with 9 and 10 atoms in the bridge reduce the α-helical content by 21% and 12% respectively, possibly because they are too short to permit formation of an unstrained α-helix [85]. The 11- and 12-membered bridges both exert a stabilizing effect. The effect is greatest for the 11-membered bridge, for which the α-helicity compared to the parent C-peptide sequence increases by 44% [85].

Protease lability is generally regarded as an inherent problem with peptide pharmaceuticals. However, processing by proteases require that the substrate binds in an extended conformation. If a peptide can be stabilized in a helical conformation proteases will not be able to recognize and cleave it. In support of this hypothesis Verdine and co-workers have presented convincing data on the proteolytic stability of a hydrogenated version **96** of their *i*→*i+7* (m = 11) tethered peptide **95** compared to its acyclic diolefinic precursor **94** and the native C-peptide sequence from RNAse A. The rate constant for trypsin digestion is an impressive 41-fold smaller for the stapled peptide than for the native sequence [85].

In a further successful application of *i*→*i+7* hydrocarbon stapling Walensky and Verdine stabilized the 15 residue long transactivation domain of the protein p53 in an α-helical conformation [112]. p53 is a transcription factor which induces cell cycle arrest and programmed cell death (apoptosis). Verdine and Walensky’s stapled peptides compete with p53 for the E3 ubiquitin ligase hDM2, which, when bound to p53, neutralizes its apoptotic effect. This has the effect of restoring p53 activity and inducing cell death – with applications in cancer therapy [112]. However, despite the fact that the transactivation domain of p53 adopts an α-helical conformation when bound to hDM2 [113], recent studies suggest that there might not be a direct correlation between α-helicity and activity for this particular class of stapled peptides [113,114]. Whether we will see any stapled peptides on the market soon remains uncertain, but Verdine and Walensky’s methodology has attracted significant interest from the pharmaceutical industry recently [115].

## 29. Conclusions

In this article we have attempted to review the current state of knowledge about the structural and pharmacological effects of RCM in peptidic systems. From the many and diverse examples of RCM in peptides reported, of which we have tried to make a representative selection, it should be clear that cyclization can have both productive and counter-productive effects with respect to the pharmacological properties of peptides.

The usefulness of RCM for making metabolically more stable dicarba analogues of disulfide bridged peptides has been established beyond any reasonable doubt. However, in a perhaps surprisingly large number of cases RCM results in compounds with unchanged or diminished activity compared to the parent peptides. 

One of the general ideas behind introducing cyclic structures into peptidic systems is to constrain the peptides in their bioactive conformation(s), thereby paying some of the entropy penalty associated with the binding of a ligand to its receptor, thus increasing the affinities of the peptides for their targets. However, in many of the cases reviewed here the precise nature of the bioactive conformation of the parent peptide has not been known. In such cases unfortunate selection of bridge length and positioning of the bridge along the peptide backbone may lead to a *de*stabilization rather than a stabilization of the bioactive conformation – with loss of activity as result. 

However, in the cases where detailed structural information has been available on the binding geometry of a peptide ligand, e.g., from a co-crystal structure [43,80] or a high-resolution NMR structure of a peptide-receptor complex, thus making rational design of an optimal crosslink possible, RCM has proven its utility in drug design as a tool which can be used to constrain a peptide in its bioactive conformation [42,45]. 

The all-hydrocarbon stapling methodology of Verdine and Walensky [80,81,85,87,112], which not only seems to be capable of structural stabilization of α-helical peptides, increasing their receptor affinities and improving their metabolic stability, but also appears to offer a generic method for increasing the cell permeability of a peptide drug via a novel endocytic mechanism [80], is clearly a very valuable addition to chemical biology and drug design. 

It is our belief that the wealth of information which is gradually becoming available on the unique structures and pharmacological properties adopted by peptides with RCM-derived crosslinks will be of great aid in future drug design projects.
